# Supplementary material for: Sound vs. light: wing-based communication in Carboniferous insects
Source: Commun Biol. 2021 Jul 8;4:794. doi: 10.1038/s42003-021-02281-0 (PMC8266802; doi:10.1038/s42003-021-02281-0)
Supplement: Supplementary file 2 — Supplementary Information [file 42003_2021_2281_MOESM2_ESM.pdf]

Supplementary Information

Sound vs. light: Wing-based communication in Carboniferous insects

Thomas Schubnel[1,*,†], Frédéric Legendre[1,†], Patrick Roques[2], Romain Garrouste[1], Raphaël Cornette[1], Michel Perreau[3,4], Naïl Perreau[4], Laure Desutter-Grandcolas[1,†], André Nel[1,*,†]

This file includes:

Supplementary Methods

- **Material**
- **Imaging**
- **Depth map**
- **Angle calculations**

Supplementary Discussion

- **Taxonomy**
- **Phylogenetic relationships of Titanoptera**
- **Antiquity of Titanoptera**
- **Distribution of broadened zones on tegmina of Titanoptera**
- **Reflection of light on forewings of *Clatrotitan andersoni* McKeown, 1937**
- **Putative modes of communication in Titanoptera**
- **Titanoptera as diurnal insects**

Supplementary Notes

- ***Clatrotitan* McKeown, 1937 vs. *Mesotitan* Tillyard in Tillyard & Dunstan (1916)**
- **What is *Steinhardtia maryae* Jell & Lambkin, 1993?**
- **Possible crepitation in a Tettigoniidae**

References

Supplementary Methods

Material

The recently discovered outcrop at Avion in the department of Pas-de-Calais, France, is especially rich in small to very small wings mixed with thousands of plant fragments. It has provided a very diverse entomofauna that comprises Palaeodictyoptera (including larvae), Odonatoptera, Archaeorthoptera, Caloneurodea, Paoliida, Dictyoptera, and the oldest representatives of the clades Acercaria and Holometabola[1-5]. It is dated from the Moscovian (Westphalian C/D or equivalent Bolsovian/Asturian). The fossil insects were found in 'Terril N 7', which contains rocks from the slag heap of coal mines 3 and 4 of Liévin, Bolsovian (Westphalian C, 308–311 Ma, 'faisceaux de Ernestine') / Asturian (Westphalian D, 306–308 Myrs, 'veines Arago, Dusouich, Marthe'; Bruno Vallois 2013 pers. comm.). The new fossil (MNHN.F.A70111) was collected in sampled rocks, using systematic observation of the pieces of rocks under a lens. The fossil is stored in the collection of the Muséum national d'Histoire naturelle, Paris (MNHN), France.

Imaging

The fossils were studied in a dry state using Olympus SZX-9 and Nikon SMZ 1500 stereomicroscopes. Photographs were taken using a Nikon D800 digital camera with Nikon AF-S Micro NIKKOR 60mm f/2.8G ED. Original photographs were processed using the image-editing software Adobe Photoshop CS. We follow the wing venation nomenclature of Archaeorthoptera proposed by [6-7], later confirmed in a recent study[8]. The venational symbols used are specified as follows: symbols in capitals denote the longitudinal veins (C costa, ScP: subcostal posterior, RA/RP: radial anterior/posterior, M: medial, CuA/CuP: cubital anterior/posterior).

Depth map

In order to visualize and quantify the slight relief of the fossils, we proceeded as follows: a Principal component analysis (PCA) was performed on the coordinates of the wing point cloud. By definition, the two first axes of the PCA show the wing on its longest length and then its widest width. Then, these first two axes were colored according to the 'z' axis illustrating and quantifying the depth map. The PCA and the visualizations have been done using R version 3.5.1. R Core Team[9].

Angle calculations

After defining the area to be measured, the mesh was cut to create a profile using the software © Geomagic Wrap and then an oriented image (maximizing 'x' and 'y') of the profile was exported. The angles of interest of the profile have been realized using the TpsDig 2 software[10].

Supplementary Discussion

Taxonomy

Since all taxa are monotypic, the included species and genus are automatically herein designated as the type species and type genus for the associated generic and familial names.

Class Insecta Linné, 1758

Order Titanoptera Sharov, 1968

Family Theiatitanidae Schubnel, Roques & Nel, fam. nov.

Theiatitan azari Schubnel, Roques & Nel, gen. & sp. nov.

Etymology. The generic name refers to Theia, the Titanide of light, while 'titan' refers to the common suffix of the Titanoptera. The gender of the name is masculine. The specific epithet refers to our friend and colleague Pr. Dany Azar.

Material. Holotype MNHN.F.A70111 (Avion 37), sex unknown, imprint and counterimprint of mid part of a wing, collected by Patrick Roques; MNHN, Paris, France.

Type locality. 'Terril N 7', containing rocks from the slag heap of coal mines 3 and 4 of Liévin, Avion, Pas-de-Calais, France.

Type horizon. Moscovian (Westphalian C/D or equivalent Bolsovian/Asturian), Carboniferous.

Diagnosis. Forewing venation characters only. Wing tegminized, with small spines on longitudinal veins; main veins not S-shaped; RP long and straight; broad areas between the RP and M, the branches of M, M and CuA, and between CuA and CuP/posterior wing margin; numerous concave veinlets perpendicular to main veins in these areas, separating cells each with a convex surface; part of CuA long basal of its fusion with CuPaα; distal part of CuA very long and distally parallel to posterior wing margin; free branch(es) of CuPaα and CuPaβ short, not reaching mid part of wing (not present in this part of wing).

Description (Fig. 1, Supplementary Fig. 1). Mid part of a (fore?) wing, relatively broad, preserved part 30.6 mm long, wing ca. 11.3 mm wide, wing apparently tegminized with small spines on all veins (allowing to accurately follow the main veins); costal margin basally deformed; a vein (C or ScA?) closely parallel to costal margin at wing base; ScP parallel to costal margin, partly preserved, with its basal part superposed to radial vein, not fused with it; a series of rather long crossveins between it and R; a series of rather long crossveins between anterior wing margin and ScP; convex R+M+CuA forked into convex R and convex M+CuA 3.7 mm from wing base; R forked into convex RA and concave RP 5.8 mm distally; RP straight and simple in its preserved part; area between RP and M broad, 1.9 mm wide, with a series of long parallel concave veinlets perpendicular to them; distal common stem M+CuA short, forked 2.7 mm distal of its base; stem of M 6.2 mm long, M forked into two branches enclosing a long area, 9.2 mm long and 1.7 mm wide, with a series of long parallel concave veinlets

perpendicular to branches of M, these being fused again distally to separate again more distally; area between M and CuA broad, 2.0 mm wide, with a series of long parallel veinlets perpendicular to M and CuA; CuA very convex, not preserved in its median part, with three veinlets ending in its preserved basal part, distal part of CuA(+CuPaα) long, parallel with posterior wing margin and emitting two weakly defined branches; two rows of cells between CuA and posterior wing margin, area between CuA and posterior wing margin 2.5 mm wide; main part of CuP and anal area not preserved, except for the basal part of CuPa that follows the posterior brake of the wing (visible thanks to a series of aligned small spines), part of CuA basal of its fusion with a CuPaα; the shape of mid part of wing shows that the free branches of CuPa were not very long and not parallel to posterior wing margin.

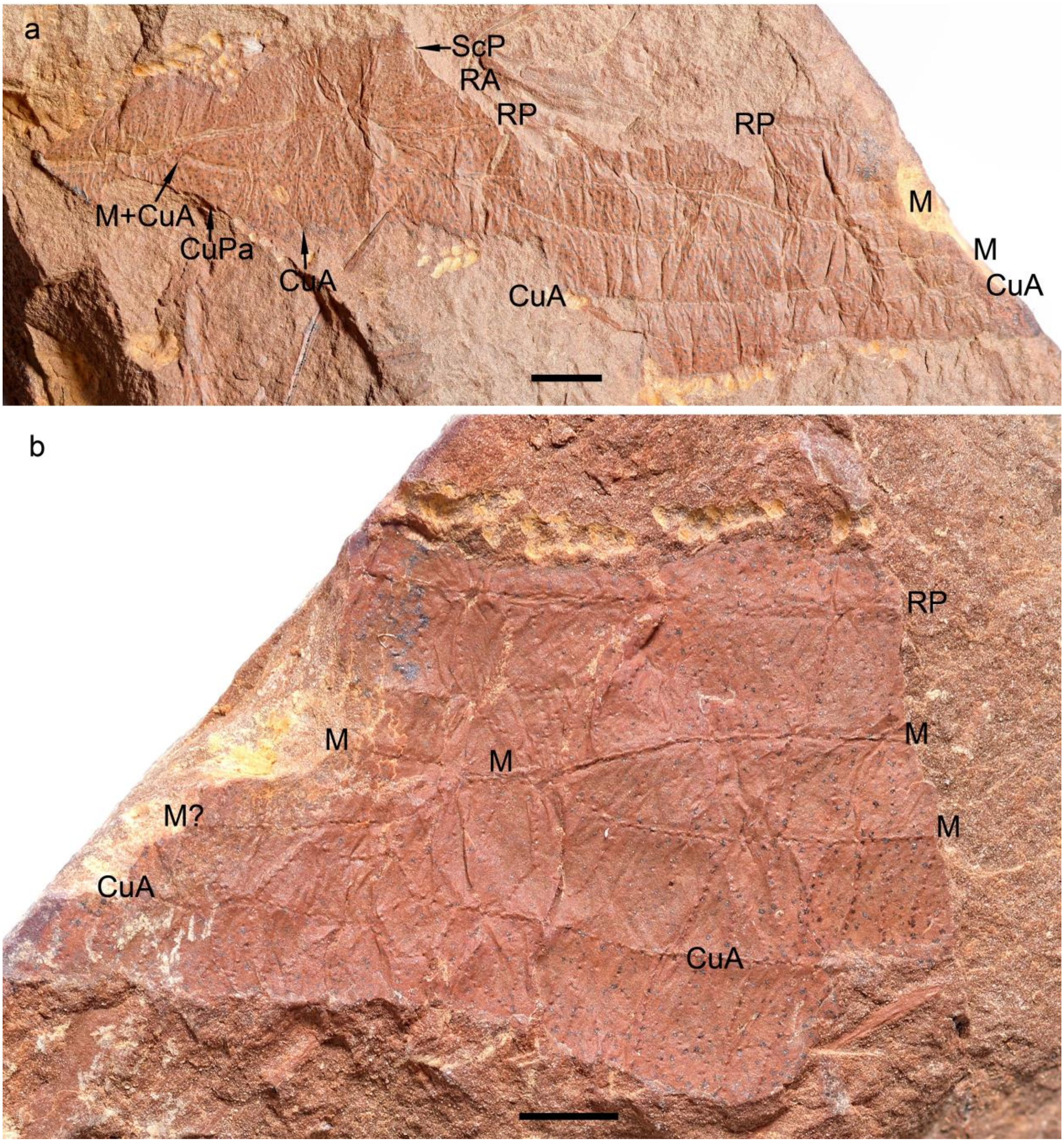

Supplementary Figure 1 | *Theiatitan azari* sp. nov., holotype MNHN.F.A70111. (**a**) general view of imprint. (**b**) counterimprint of mid part of wing. Abbreviations: C costa, ScP: subcostal posterior, RA/RP: radial anterior/posterior, M: medial, CuA/CuP: cubital anterior/posterior. Scale bars: 2 mm.

Discussion. This fossil corresponds to a forewing because of the broad area between R and ScP, with numerous transverse veinlets, the general shape of main veins, and its tegminisation.

This wing could be attributed to the Acercaria or to the Archaeorthoptera because of the basal fusion of R, M, and CuA[7-11]. Among the Acercaria, only the Hemiptera Auchenorrhyncha could have similar sclerotized forewings and complex venation, but they differ from *Theiatitan* in the vein ScP basally fused to R+M+CuA, the absence of a vein parallel to costal margin at wing base but not appressed to it, and the presence of a fork of CuA (areola postica), which is reduced in few groups that are dated from the Cretaceous to recent (e.g. Issidae)[12-13]. The very particular shape of areas between RP, M and its branches, M and CuA, and between CuA and posterior wing margin, all broadened, distally closed, and with long transverse veins perpendicular to main veins, can be found in the Titanoptera only[6,14]. Even if some Cicadomorpha (e.g. Cicadoprosbolidae, Tettigarctidae, etc.) also have closed cells in mid part of forewing, they also have an areola postica, no ScP separated from radius, no vein ScA, and the broadened areas in mid wing are free of crossveins, unlike in *Theiatitan*. On the other side, the very long and simple CuA parallel to M is found in the Titanoptera. Thus we attribute this fossil to this clade.

Theiatitan differs from all other known Titanoptera in its broadened areas, especially that between CuA and CuP/wing margin, which support its attribution to a new family: it can be separated from the Gigatinidae Sharov, 1968 in the presence of a broad area between the branches of M; the Paratitanidae Sharov, 1968 differ from *Theiatitan* in the narrower areas between the branches of M and between M and CuA, a shorter CuA basal of its fusion, plus a longer CuPa parallel to posterior wing margin (this vein is certainly shorter in *Theiatitan* than in the Paratitanidae as there is no trace of it in the preserved part of wing between CuA and posterior wing margin); in the Mesotitanidae Tillyard, 1925, the veinlets in these broadened areas are alternatively concave and convex, unlike in *Theiatitan*, and CuPa is longer and parallel to the posterior wing margin, reaching mid part of wing. The Permian Deinotitanidae Gorochov, 2007 have the main longitudinal veins S-shaped[14-19], but they do not belong to the Titanoptera (see below – **Antiquity of the Titanoptera**). *Theiatitan* also differs from all the other

Titanoptera in the distally closed area between the two branches of M, instead of ending in wing margin, but this structure is possibly an aberration in the venation.

Phylogenetic relationships of Titanoptera

These insects are currently placed in the Archaeorthoptera[6]. Aristov[20] put them in his 'Orthopteridea', but he considered the Geraridae as 'ancestors' of the Orthoptera, Phasmatodea, and Titanoptera. As the extant Phasmatodea are very far from the extant Orthoptera in the most recent molecular phylogenies[21-23], this last hypothesis is rather unlikely. Gorochov[12-13] suggested that the extant Mantophasmatodea are 'descents' of the Titanoptera, but the titanopteran wing venation shows the main synapomorphies of the Archaeorthoptera ('orthopteroid' insects)[6], while the apterous Mantophasmatodea are currently considered as sister clade of the apterous Grylloblattodea, very far from the Orthoptera.

Béthoux[6] proposed to consider the Permian archaeorthopteran 'Tcholmanvissiidae' Zalessky, 1934 and Tettoedischiidae Gorochov, 1987 as basal groups of the Titanoptera. More precisely he considered that the family Tcholmanvissiidae is a clade that includes the order Titanoptera. These families have relatively narrower areas between RP and M, branches of M, and M and CuA than the titanopteran taxa, including *Theiatitan*. The relationships between the Titanoptera and the Tcholmanvissiidae remain controversial[24-25].

Antiquity of Titanoptera

The present discovery of a Titanoptera in the late Carboniferous is unsurprising. Gorochov[16] supposed that 'the Mesozoic evolutionary stage of the superorder Orthopteroidea possibly started not in the Early Triassic, but rather in the Late Permian' because of his discoveries of Permian Titanoptera—see also[19,26]. The Permian taxa that Gorochov placed into the Titanoptera are, however, not Titanoptera, as shown by recent discoveries of better preserved fossils from

the Middle Permian of China, which are clearly related to them and belong to ensiferan Orthoptera (Huang et al., in prep.). Nevertheless, the present discovery of *Theiatitan* shows that, if Titanoptera greatly diversified during the Triassic, they are clearly much older. Also, the recent discoveries in Germany of Early Permian very large grasping insect fore legs with very strong spines, very similar to those of the Triassic Titanoptera, support the presence of true Titanoptera during the Permian (Harald Stapf, pers. comm.).

Distribution of broadened zones on tegmina of Titanoptera

In *Theiatitan*, the areas between RP, M, CuA and CuP are concerned; they contain a series of relatively irregular intercalary veinlets separating large cells. In *Minititan zherichini*, only the area between the branches of M is significantly broadened with regular large cells[23]. In *Nanotitan magnificus* Sharov, 1968 and *Gigatitan vulgaris*, only the area between M and CuA is significantly broadened, but it is subdivided into several smaller cells by an irregular network of weaker crossveins in *Nanotitan* while this network of cells is better organized, being subdivided in two rows by a zigzagged vein in *Gigatitan* (Fig. 2, Supplementary Fig. 2)[14]. Lastly, unlike in *Theiatitan*, the three areas between RP, branches of M, and CuA (but not between CuA and CuP) are broadened in *Mesotitanodes tillyardi* Sharov, 1968, *Paratitan ovalis* Sharov, 1968, *Paratitan venosus* Gorochov, 2003, and *Clatrotitan andersoni*. All have a regular series of straight veinlets alternatively concave and convex defining very particular large cells, each subdivided into two parts of different oblique orientations.

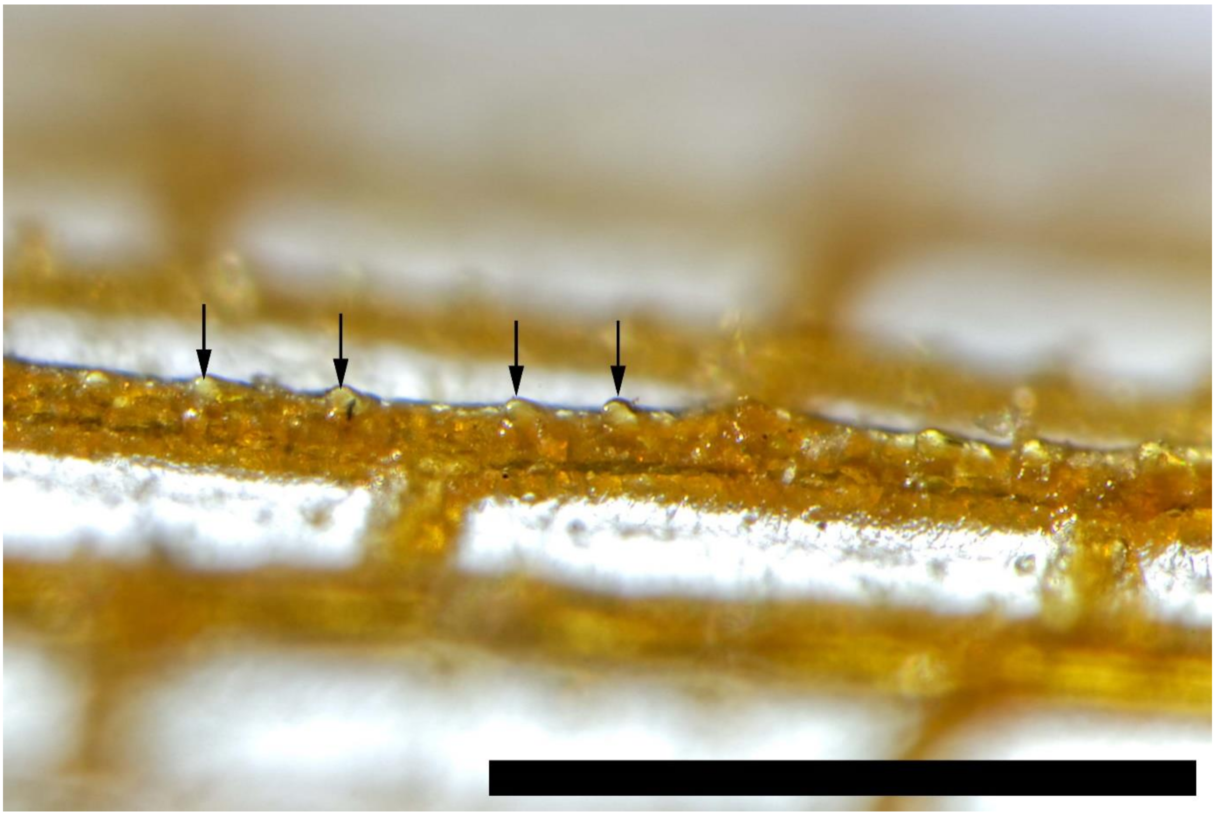

Supplementary Figure 2 | *Tropidopola cylindrica*, photograph of vein spines on RP and M. Scale bar: 0.5 mm.

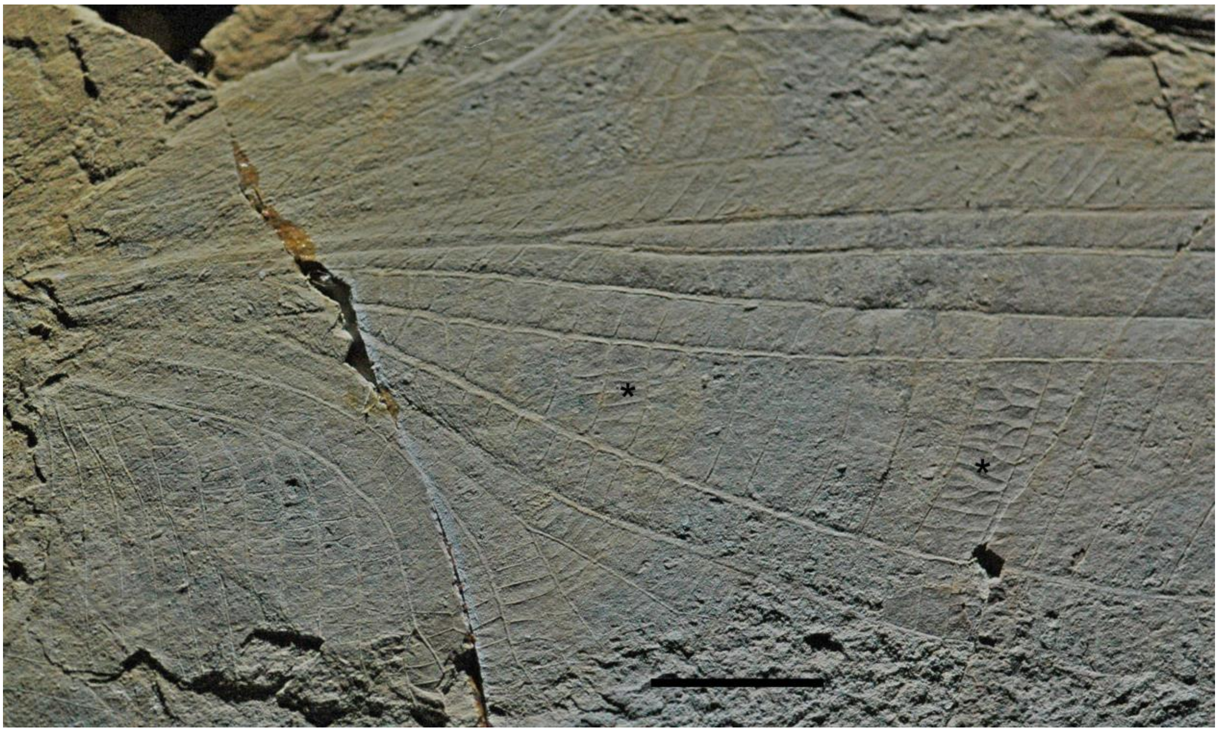

Supplementary Figure 3 | *Gigatitan vulgaris* Sharov, 1968, PIN 2555/1541. Forewing, (*) large cells subdivided into a net of small cells with different orientations. Scale bar: 10 mm.

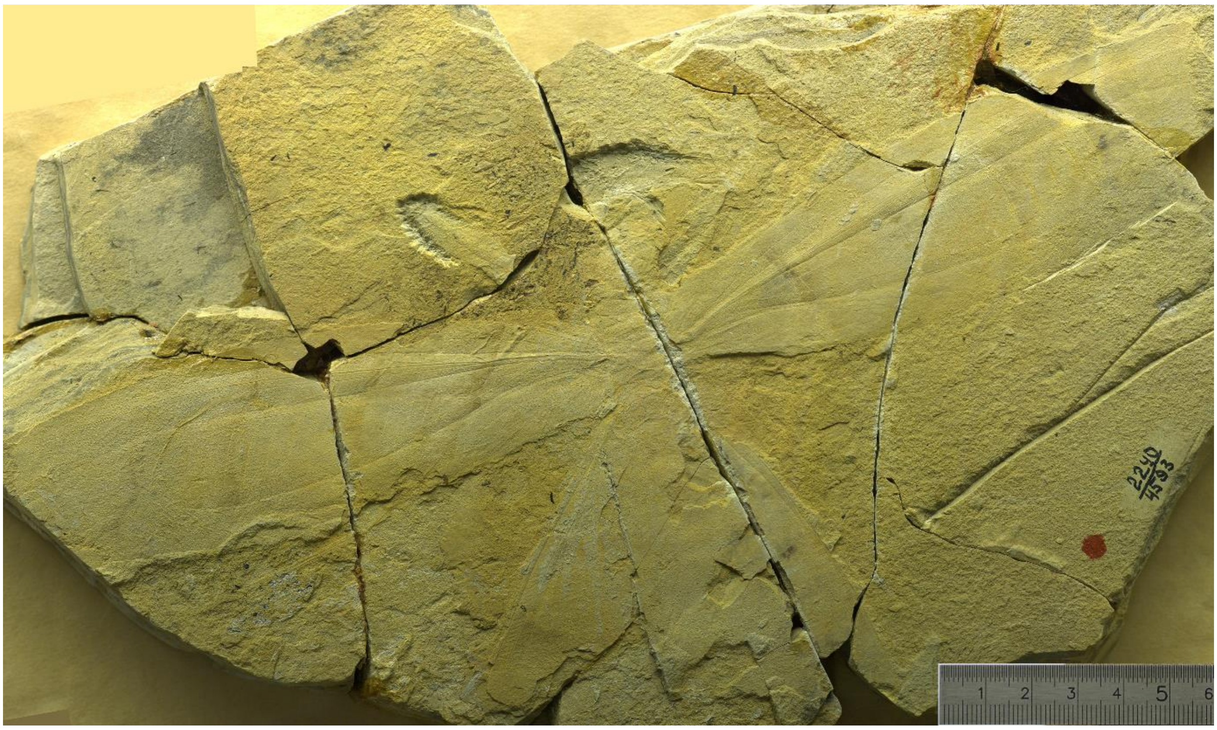

Supplementary Figure 4 | *Gigatitan vulgaris* Sharov, 1968, PIN 2240/4593. Nearly complete body with wings and fore legs.

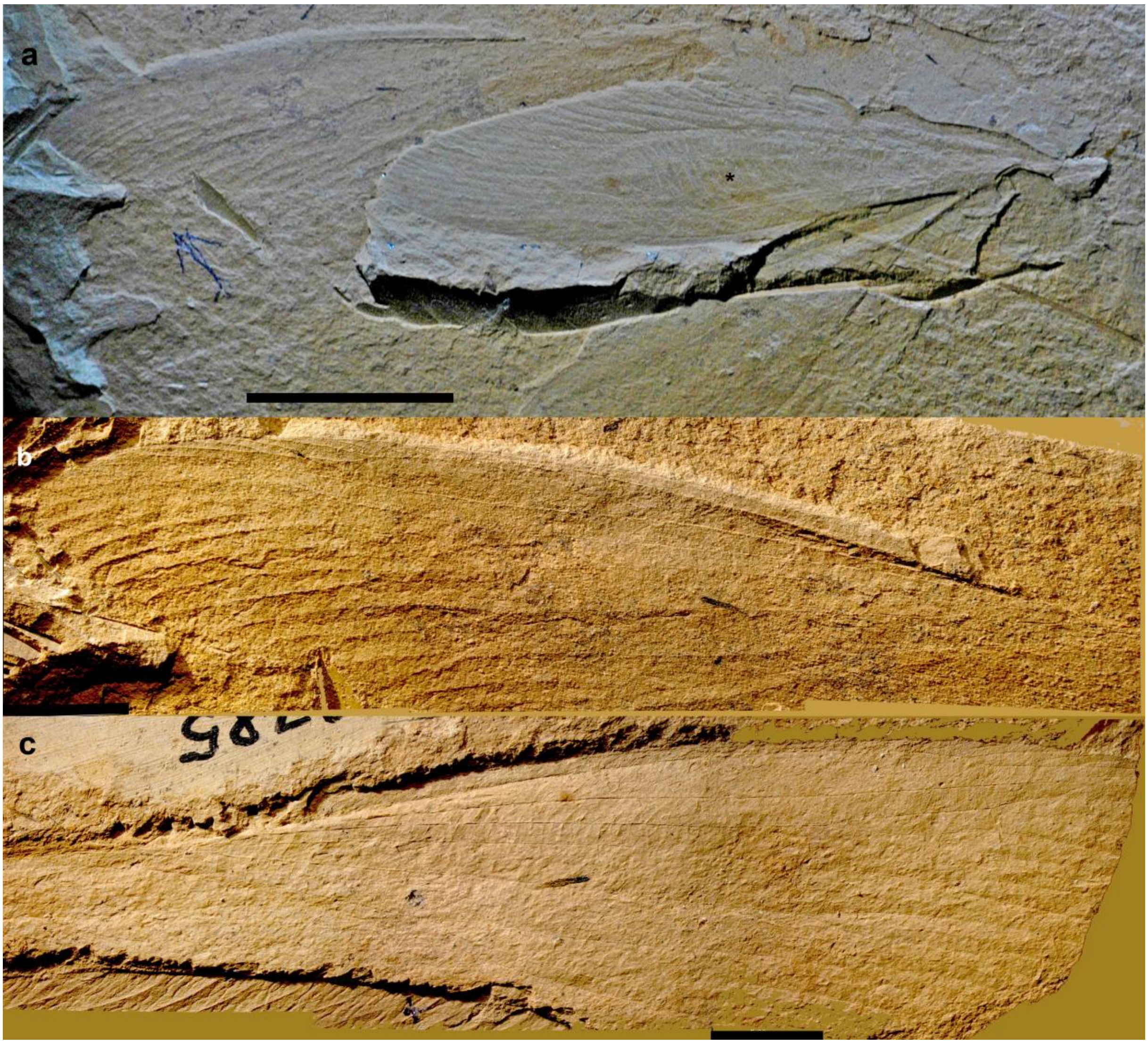

Supplementary Figure 5 | *Mesotitanodes similis* (Sharov, 1968), PIN 2785/2029. (**a**) general view, (*)forewing specialized zone. (**b**) hindwing imprint. (**c**) hindwing counterimprint. Note that, contrary to forewing, the hind wings do not show any specialized zone or vein. Scale bars: 5 mm.

Reflection of light on the forewings of *Clatrotitan andersoni* McKeown, 1937 (Supplementary Tables 1-2; Supplementary Fig. 6; Supplementary Movies 1-2)

Calculations are made within the following hypothesis: the cells of the specialized structures observed on the wings of *Clatrotitan* are asymmetric, the area of one side being approximately 2/3 to 1/2 times the area of the other; the angle between the two sides is constant (~ 148°); no

global tilt angle (the external edges of the cell walls are at the same distance of the average plane of the wing).

Reminder: the incidence angle of a light ray on a plan is the angle between the light ray and the normal to the plan.

Notations:

α: incidence angle on the average plan of the wing.

θ: angle at the vertex of a cell. The experimental value is θ ~ 145-150°, we take the mean value ~148°.

β1: angle between the plane of the wing and the plane of the small side of the cell.

β2: angle between the plane of the wing and the plane of the large side of the cell.

Necessarily β1+β2 = 180°–148° = 32°. From the experimental ratio of areas of the sides of cells, we have sin(β2)/sin(β1) ~ 2/3 to 1/2. We retain the intermediate value of 0.608 which makes β1 = 20° and β2 = 12° as in supplementary Fig. 6 and in the animation of supplementary file 6.

Angles are counted from left (negative values) to right (positive values) and beams reflected on the small side of the cell (left side in Supplementary Fig. 6) and on the large side of the cell (right side in Supplementary Fig. 6) are denoted respectively '1' and '2'.

Calculations:

Detailed calculations are given for an incidence on the small side of the cell and are illustrated in Supplementary Fig. 6. For each general expression, the value corresponding to the experiment conditions are given between square brackets. Starting from α = 0 with α < 0, two situations appear successively:

* For –π/2+2β1+β2 [–38°] < α < 0, the beam reflected on the small side (emergent beam 1) emerges directly without meeting the large cell (Supplementary Fig. 6, top):

– Incidence angle on the average plan of the wing: α

– Incidence angle on the average plan of the small side of the cell: $\alpha - \beta 1$

– Reflected angle on the average plan of the small side of the cell: $-\alpha + \beta 1$ (Snell-Descartes rule)

– Emergence angle on the average plan of the wing: $-\alpha + 2\beta 1$ [$-\alpha + 40°$]

* For $\alpha < -\pi/2 + 2\beta 1 + \beta 2$, the reflected beam on the small side undergoes a second reflection on the large side with an incidence angle $\alpha' = -\pi - \alpha + 2\beta 1$ (Supplementary Fig. 6 bottom). For this second reflection (emergent beam 1'):

– Incidence angle on the average plan of the wing: $\alpha' = -\pi - \alpha + 2\beta 1$

– Incidence angle on the average plan of the large side of the cell: $\alpha' + \beta 2 = -\pi - \alpha + 2\beta 1 + \beta 2$

– Emergence angle on the average plan of the large side of the cell: $-\alpha' - \beta 2 = \pi + \alpha - 2\beta 1 - \beta 2$ (Snell-Descartes rule)

– Emergence angle on the average plan of the wing: $-\alpha' - 2\beta 2 = \pi + \alpha - 2(\beta 1 + \beta 2)$ [$\alpha + 116°$]

As α decreases, beam 1' gradually substitutes to beam 1 from $\alpha = -\pi/2 + 2\beta 1 + \beta 2$ [$-38°$] to $\alpha = -\pi/2 + 2\beta 1$ [$-50°$] where beam 1 vanishes. The beam 1' makes a fixed angle $\pi - 2(\beta 1 + \beta 2)$ [$116°$] with the direction of incidence, independently of the incidence angle α.

When $\alpha > 0$, the single beam 1 occurs, 1' does not exist. It gradually vanishes when $\alpha > \pi/2 - \beta 2$ [$78°$] since the incidence becomes larger than the angle of the large side of the cell (grey columns of the Supplementary Tables).

For incidences on the large side of the cell, the situation is symmetric, with the changes $\beta 2 \leftrightarrow -\beta 1$. Starting from $\alpha = 0$ with $\alpha > 0$, beam 2 emerges with an angle $-\alpha - 2\beta 2$ [$-\alpha - 24°$] and beam 2' with an angle $-\pi + \alpha + 2(\beta 1 + \beta 2)$ [$\alpha - 116°$] (all angles reported to the average surface of the wing). As α increases, beam 2' gradually substitutes to beam 2 from $\alpha = \pi/2 - \beta 1 - 2\beta 2$ [$46°$] to $\alpha = \pi/2 - 2\beta 2$ [$66°$] where beam 2 vanishes. The beam 2' makes a fixed angle $-\pi + 2(\beta 1 + \beta 2)$ [$-116°$] with the direction of incidence, independently of the incidence angle α. When $\alpha < 0$, the single beam 2 occurs, 2' does not exist. It gradually vanishes when $\alpha < -\pi/2 + \beta 1$ [$-70°$] since the incidence becomes larger than the angle of the small side of the cell (grey columns in tables).

For the limits values of α: $\pi/2-\beta2$ [78°] and $-\pi/2+\beta1$ [–70°] respectively, beams 1' and 2 on one hand and beams 1 and 2' on the other hand emerge both with the same angle (but respectively 2 and 1 are vanishing). Above $\pi/2-\beta2$ [78°] and below $-\pi/2+\beta1$ [–70°] respectively (grey columns in tables), a single side of the cell still remains enlightened, beams 1 and 2 vanish until $\alpha = \pi/2$ and $\alpha = -\pi/2$ respectively.

Incidence/wing α	$-\pi/2$	$-\pi/2+\beta_1$	$-\pi/2+2\beta_1$	$-\pi/2+2\beta_1+\beta_2$	0	$\pi/2-\beta_1-2\beta_2$	$\pi/2-2\beta_2$	$\pi/2-\beta_2$	$\pi/2$
Beam 1/wing			$\pi/2$		$-\alpha+2\beta_1$			$-\pi/2+2\beta_1+\beta_2$	$-\pi/2+2\beta_1$
Beam 1/incidence			$\pi-2\beta_1$		$-2(\alpha-\beta_1)$			$-\pi+2(\beta_1+\beta_2)$	$-\pi+2\beta_1$
Beam 1'/wing		$\pi/2-\beta_1-2\beta_2$	$\pi+\alpha-2(\beta_1+\beta_2)$	$\pi/2-\beta_2$					
Beam 1'/incidence		$\pi-2(\beta_1+\beta_2)$ - - - - - - - - - -		$\pi-2(\beta_1+\beta_2)$					
Beam 2/wing	$\pi/2-2\beta_2$	$\pi/2-\beta_1-2\beta_2$			$-\alpha-2\beta_2$		$-\pi/2$		
Beam 2/incidence	$\pi-2\beta_2$	$\pi-2(\beta_1+\beta_2)$			$-2(\alpha+\beta_2)$		$-\pi+2\beta_2$		
Beam 2'/wing						$-\pi/2+\beta_1$	$-\pi+\alpha+2\beta_1+2\beta_2$	$-\pi/2+2\beta_1+\beta_2$	
Beam 2'/incidence						$-\pi+2(\beta_1+\beta_2)$ - - - - - - - - - -		$-\pi+2(\beta_1+\beta_2)$	

Supplementary Table 1 | Incident and emergent angles reported to normal to average plan of wing and to incident direction. Color codes are the same as in supplementary Fig. 5: red for the incident beam, green for the emergent beams 1 and 1', blue for the emergent beams 2 and 2'. The intensity of colors maps the intensity of light beams.

Incidence/wing α	–90°	–70°	–50°	–38°	0	46°	66°	78°	90°
Beam 1/wing			90°		$-\alpha$+40°			–38°	–50°
Beam 1/wing			140°		-2α+40°			–116°	–140°
Beam 1'/wing		116° - - - - - - - - - - - - - - - -		116°					
Beam 1'/incidence		46°	α+116°	78°					
Beam 2/wing	66°	46°			$-\alpha$–24°		–90°		
Beam 2/incidence	156°	116°			-2α–24°		–156°		
Beam 2'/wing						–70°	α– 116°	–38°	
Beam 2'/incidence						–116° - - - - - - - - - - - - - - -		–116°	

Supplementary Table 2 | Incident and emergent angles reported to normal to average plan of wing and to incident direction. Same as Supplementary Table 1, for $\beta1 = 20°$ and $\beta2 = 12°$,

corresponding to approximate experimental values, as illustrated in Supplementary Fig. 6 and in animation of Supplementary Movie 1.

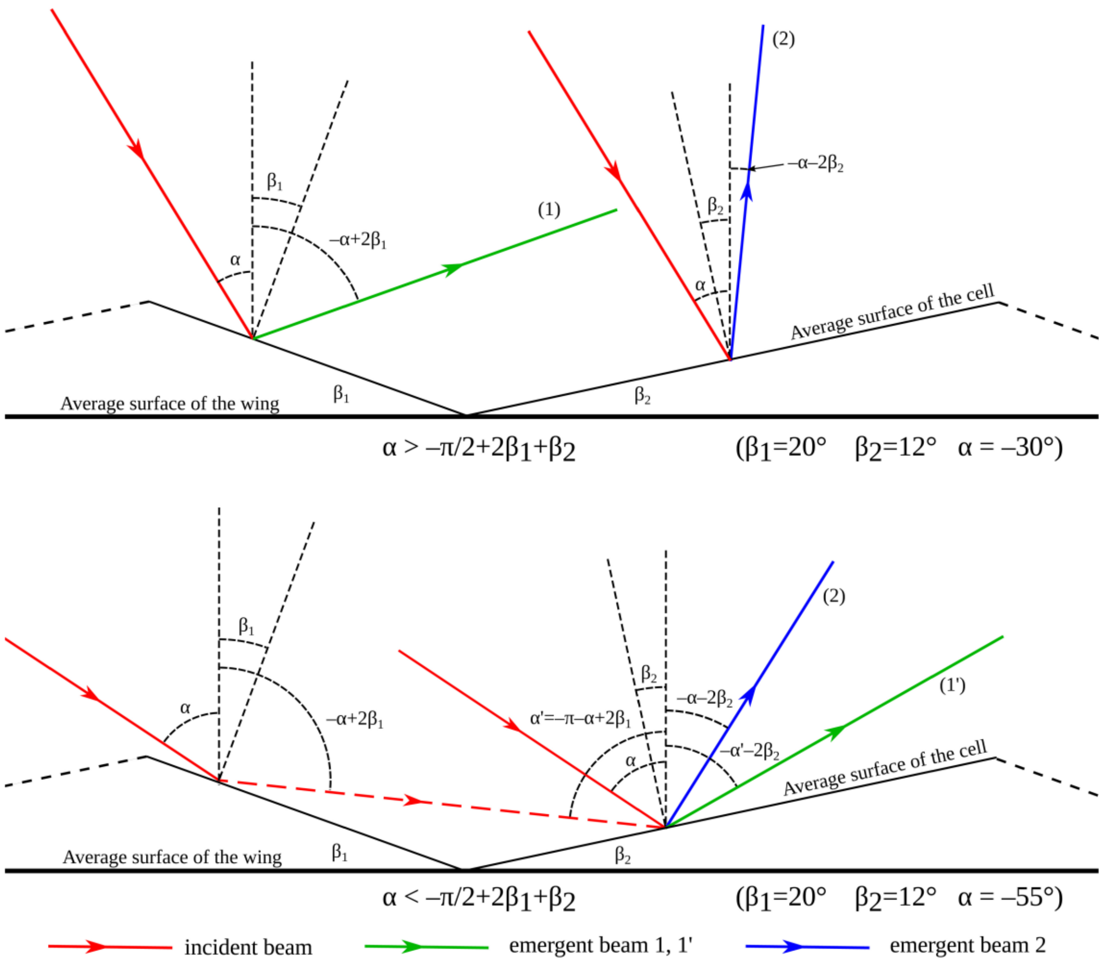

Supplementary Figure 6 | Emergent beams 1 and 2. Single reflection (top) and 1' with a double reflection (bottom).

Putative modes of communication in Titanoptera

Crepitation hypothesis supported by broadened areas with large cells in forewing.

Stridulation hypothesis supported by aligned teeth on one specialized longitudinal vein (*Theiatitan* has small spines on all its veins, none of them being devoted to a special function).

Light flashes hypothesis supported by regular pattern of two sets cells with different orientations in a broadened area.

Late Carboniferous

Theiatitan azari gen. et sp. nov.: crepitation

Permian

Permotitan vladimiri: no specialized structure, dubious attribution to Titanoptera

Deinotitan orenburgensis: stridulation structure of Ensiferan type, not a Titanoptera (Huang et al., in prep.)

Monstrotitan monstrosus: no specialized structure on forewing, hind wing with strong modifications of the venation, looking like a stridulatory structure (resonator); possibly not a Titanoptera but a Caloneurodea (Huang et al., in prep.)

Triassic

Gigatitan vulgaris: crepitation

Gigatitan ovatus: crepitation

Gigatitan similis: crepitation

Prototitan primitivus: crepitation

Prototitan sharovi: crepitation

Ootitan curtis: crepitation, light flashes?

Nanotitan extensus: crepitation, light flashes?

Nanotitan magnificus: crepitation, light flashes?

Ultratitan superior: crepitation, light flashes?

Mesotitanodes tillyardi: crepitation, light flashes?

Paratitan ovalis: crepitation?, light flashes?

Mesotitanodes similis: light flashes, crepitation?

Microtitan zherichini: light flashes, crepitation?

Clatrotitan andersoni: light flashes

Mesotitan scullyi: light flashes (for synonymy between *Clatrotitan* and *Mesotitan*, see Supplementary Notes)

Paratitan libelluloides: light flashes

Paratitan longispeculum: light flashes

Paratitan reductus: light flashes

Paratitan venosus: light flashes

Paratitan latispeculum: light flashes

Paratitan bispeculum: light flashes

Paratitan modestus: light flashes

Paratitan reliquia: unknown (hind wing only)

Steinhardtia maryae Jell & Lambkin, 1993 (not an insect Titanoptera, see Supplementary Notes)

Titanoptera as diurnal insects

According to Sharov[14], the Titanoptera were diurnal on the basis of the presence of dark transverse stripes (disruptive pattern of coloration?) on the wings of *Gigatitan vulgaris*. For instance, similar stripes are present in the diurnal mantis *Blepharopsis mendica*. This hypothesis would be congruent with a diurnal production of flashes of light. Nevertheless some extant insects with stripes are nocturnal, e.g., the stick insect *Phasma gigas* has also broad dark stripes on wings but it is active by night; these stripes could have a function of frightening the predator when the insect opens its large wings, or be cryptic at rest. It is also possible that Titanoptera included both diurnal and nocturnal species, as Orthoptera do for example.

Supplementary Notes

Clatrotitan McKeown, 1937 vs. _Mesotitan_ Tillyard in Tillyard and Dunstan (1916)

Tillyard & Dunstan[18] described the fossil genus *Mesotitan* (type species *Mesotitan giganteus* Tillyard, 1916) on the basis of a very poorly preserved fossil[26]. Later Tillyard[17] described a second species *Mesotitan scullyi* on the basis of female tegmina. McKeown[27] created the new genus and species *Clatrotitan andersoni*. Riek[28] indicated that *Mesotitan giganteus* is 'a quite distinct genus [from *Clathrotitan* (sic)] most probably referable to the Homoptera'. Thus he transferred *Mesotitan scullyi* into the genus *Clatrotitan*. Carpenter[13] synonymized *Clatrotitan* with *Mesotitan* and *C. andersoni* with *M. scullyi* without argument. Jell[29] restored *Clatrotitan* but maintained the synonymy between *C. andersoni* with *C. scullyi*. An issue is that *C. andersoni* is based on tegmina with expanded areas in mid parts, while *C. scullyi* is based on fragmentary wings without such structures [interpreted as hind wings after Sharov[14] or as female forewings after Zeuner[30]], so that this synonymy is uncertain. Grimaldi & Engel[31] figured the type forewing of *C. andersoni*. Lastly Béthoux[6] named '*giganteus* Tillyard, 1916' the counterimprints of the type wings of *C. andersoni*.

We propose to keep the two genera *Clatrotitan* and *Mesotitan* separated, to include in *Mesotitan* only the type species *M. giganteus*, to consider that the other *Mesotitan* species belong to the genus *Clatrotitan*, and lastly to restore the separation between *C. andersoni* and *C. scullyi* because there is no formal proof of the common identity of these two species. We then consider the following new combination *Clatrotitan scullyi* n. comb.

What is _Steinhardtia maryae_ Jell & Lambkin, 1993?

This genus and species were originally attributed to the Titanoptera[32], refigured by Jell[29], and cited by Grimaldi & Engel[31]. This fossil does not show the venational structures of the order Titanoptera. Its alleged vein CuA is not fused with MP; there is no crossveins between the main veins; there is no vein AA1; the alleged vein AA2 is basally fused with Cu, instead of being

free; the alleged vein M is basally fused for a long distance with R; there are numerous anastomoses between the main veins, unlike Titanoptera; the alleged 'strut' that would connect CuA with M is not oblique but nearly at right angle with the two veins; there are numerous divisions of main veins along the posterior wing margin, unlike in Titanoptera. All the photographs of this wing clearly show that it is a broken leaf of plant, probably a fern. Thus, it has to be excluded from Titanoptera and Insecta. It is a Plantae *incertae sedis* nov. sit.

Possible crepitation in a Tettigoniidae

The males of the tettigoniid *Segestidea queenslandica* Rentz et al., 2006 have a genuine tettigoniid stridulating apparatus in the basal part of the forewing cubito-anal zone, but also quite broadened zones in mid part of forewings, limited by ScP and C and by branches of M, with long perpendicular veinlets that define large transverse cells, themselves subdivided into a net of small cells, very similar to the 'specialized' structures of the Titanoptera *Gigatitan vulgaris*[33]. The highly specialized forewing structures of *Segestidea queenslandica* are certainly not used for stridulation but, maybe, to emit crepitate sounds, which could complement usual stridulation: Tettigonids actually keep their forewings in a roof-position above the body while singing, contrary to crickets that raised their forewings high above the body and largely open them to stridulate. It is a nocturnal species. The females do not have so developed zones in their tegmina. Unfortunately, the courtship behavior of *Segestidea queenslandica* remains unknown[34].

References

1. Nel, A., Roques, P., Nel, P., Prokin, A. A., Bourgoin, T., Prokop, J., Szwedo, J., Azar, D., Desutter-Grandcolas, L., Wappler, T. Garrouste, R., Coty, D., Huang, D-Y., Engel,

M. & Kirejtshuk, A. G. The earliest known holometabolous insects. *Nature* **503**, 257–261 (2013).

2. Nel, A., Roques, P., Prokop, J. & Garrouste, R. A new, extraordinary 'damselfly-like' Odonatoptera from the Pennsylvanian of the Avion locality in Pas-de-Calais, France (Insecta: 'Exopterygota'). *Alcheringa* **43**, 241–245 (2018).
3. Prokop, J., Tippeltová, S., Roques, P. & Nel, A. A new genus and species of Breyeriidae and wings of immature stages from the Upper Carboniferous, Nord-Pas-de-Calais, France (Insecta: Palaeodictyoptera). *Insect Systematics & Evolution* **44**, 117–128 (2013).
4. Prokop, J., Roques, P. & Nel, A. New non-holometabolous insects from Pennsylvanian of Avion locality in Pas-de-Calais, France (Insecta: 'Exopterygota'). *Alcheringa* **38**, 155–169 (2014).
5. Coty, D., Háva, J., Prokop, J., Roques, P. & Nel, A. New archaeorthopteran insects from the Late Carboniferous of the Nord and Pas-de-Calais basins in northern France (Insecta: Cnemidolestodea, Panorthoptera). *Zootaxa* **3878**, 462–470 (2014).
6. Béthoux, O. Cladotypic taxonomy applied: titanopterans are orthopterans. *Arthropod Systematics & Phylogeny* **65**, 135–156 (2007).
7. Béthoux, O. & Nel, A. Venation pattern and revision of Orthoptera *sensu nov*. and sister groups. Phylogeny of Palaeozoic and Mesozoic Orthoptera *sensu nov*. *Zootaxa* **96**, 1–88 (2002).
8. Desutter-Grandcolas, L., Jacquelin, L., Hugel, S., Boistel, R., Garrouste, R., Henrotay, M., Warren, B. H., Chintauan-Marquier, I. C., Nel, P., Grandcolas, P. & Nel, A. 3-D imaging reveals four extraordinary cases of convergent evolution of acoustic communication in crickets and allies (Insecta). *Scientific Reports* **7**, 1–8 (2017).

9. R Core Team R. A language and environment for statistical computing. R Foundation for Statistical Computing, Vienna, Austria. URL https://www.R-project.org/. (2018).
10. Rohlf, F. J. TpsDig2, digitize landmarks and outlines [software version 2.20]. State University of New York (2015).
11. Nel, A., Prokop, J., Nel, P., Grandcolas, P., Huang, D.-y., Roques, P., Guilbert, E., Dostál, O. and Szwedo, J. Traits and evolution of wing venation pattern in paraneopteran insects. *Journal of Morphology* **273**, 480–506 (2012).
12. Dworakowska, I. Main veins of the wings of Auchenorrhyncha. *Entomologische Abdhandlungen, Staatliches Museum für Tierkunde Dresden* **52**, 63–108 (1988).
13. Carpenter, F. M. Superclass Hexapoda. *In*: Moore, R. C. & Kaesler, R. L. (eds). *Treatise on Invertebrate Paleontology*. The Geological Society of America and the University of Kansas, Boulder, Colorado, (R), Arthropoda 4, **3/4**, xxii + 655 pp (1992).
14. Sharov, A. G. Filogeniya ortopteroidnykh nasekomykh. *Trudy Paleontologicheskogo Instituta, Akademiya Nauk S.S.S.R.* **118**, 1–216, Moskva. [in Russian, Translated in English in 1971: Phylogeny of the Orthopteroidea. Israel program for scientific translations, Keter Press, Jerusalem, 1–251.] (1968).
15. Gorochov, A. V. Primitive Titanoptera and early evolution of Polyneoptera. *Meeting in Memory of N.A. Cholodkovsky* **57**, 54 pp (2004).
16. Gorochov, A. V. The first representative of the suborder Mesotitanina from the Paleozoic and notes on the system and evolution of the order Titanoptera (Insecta: Polyneoptera). *Paleontological Journal* **41**, 621–625 (2007).
17. Tillyard, R. J. A new fossil insect wing from Triassic beds near Deewhy, New South Wales. *Proceedings of the Linnean Society of New South Wales* **50**, 374–377 (1925).

18. Tillyard, R.J. & Dunstan, B. Mesozoic and Tertiary insects of Queensland and New South Wales. Descriptions of the fossil insects and stratigraphical features. *Publications of the Geological Survey of Queensland* **253**, 1–63 (1916).
19. Gorochov, A. V. New taxa of the superorder Orthopteroidea from the latter half of the Permian of European Russia. *Paleontological Journal* **47**, 782–793 (2013).
20. Aristov, D. S. Palaeozoic evolution of the Insecta Gryllones. PhD Thesis, Moscow, 238 pp. (2017).
21. Misof, B., Liu, S-L., Meusemann, K., Peters, R. S., Donath, A., Mayer,C., Frandsen, P. B., Ware, J., Flouri, T., Beutel, R. G., Niehuis, O., Petersen, M., Izquierdo-Carrasco, F., Wappler, T., Rust, J., the 1KITE consortium (83 other authors), Wang, J., Kjer, K. M. & Zhou, X. Phylogenomics resolves the timing and pattern of insect evolution. *Science* **346**, 763–767 (2014).
22. Freitas, L., Mello, B. & Schrago, C. G. Multispecies coalescent analysis confirms standing phylogenetic instability in Hexapoda. *Journal of Evolutionary Biology* **31**, 1623–1631 (2018).
23. Wipfler, B., Letsch, H., Frandsen, P. B., Kapli, P., Mayer, C., Bartel, D., Buckley, T. R., Donath, A., Edgerly-Rooks, J. S., Fujita, M., Liu, S-L., Machida, R., Mashimo, Y., Misof, B., Niehuis, O., S. Peters, R. S., Petersen, M., Podsiadlowski, L., Schütte, K., Shimizu, S., Uchifune, T., Wilbrandt, J., Yan, E., Zhou, X. & Simon, S. Evolutionary history of Polyneoptera and its implications for our understanding of early winged insects. *Proceedings of the National Academy of Sciences* **116**, 3024–3029 (2019).
24. Huang, D.-y., Schubnel, T. & Nel, A. A new middle Permian orthopteran family questions the position of the order Titanoptera (Archaeorthoptera: Orthoptera). *Journal of Systematic Palaeontology* **18**, 1217–1222 (2020).

25. Béthoux, O. The nesting of titanopteran insects within tcholmanvissiids reassured and the earliest caeliferan identified: a reply to Huang et al. (2020). *Journal of Systematic Palaeontology* **18**, 1657–1668 (2020).
26. Gorochov, A. V. New and little known Mesotitanidae and Paratitanidae (Titanoptera) from the Triassic of Kyrgyzstan. *Paleontological Journal* **37**, 400–406 (2003).
27. McKeown, K. C. New fossil insect wings (Protohemiptera, family Mesotitanidae). *Records of the Australian Museum* **20**, 31–37 (1937).
28. Riek, E. F. Further Triassic insects from Brookvale, New South Wales (Orthoptera Saltatoria, Prothortoptera, Perlaria). *Records of the Australian Museum* **23**, 161–168 (1954).
29. Jell, P. A. The fossil insects of Australia. *Memoirs of the Queensland Museum* **50**, 1–124 (2004).
30. Zeuner, F. E. Fossil Orthoptera Ensifera. British Museum (Natural History), London, 1–309 (1939).
31. Grimaldi, D. A. and Engel, M. S. Evolution of the insects. Cambridge University Press, xv + 755 pp (2005).
32. Jell, P. A. & Lambkin, K. J. Middle Triassic orthopteroid (Titanoptera) insect from the Esk formation at Lake Winverhoe. *Memoirs of the Queensland Museum* **33**, 258 (1993)
33. Rentz, D. C. F., Su, Y.-N. & Ueshima, N. Studies in Australian Tettigoniidae: the mecopodine katydids. Part 2 (Orthoptera: Tettigoniidae; Mecopodinae; Sexavaini) Queensland palm katydid. *Transactions of the American Entomological Society* **132**, 229–241 (2006).
34. Rentz, D. C. F. A guide to the katydids of Australia. CSIRO Publishing, Collingwood, Victoria, i–x + 1–214 (2010).
