# Supplementary material for: Sound vs. light: wing-based communication in Carboniferous insects
Source: Commun Biol. 2021 Jul 8;4:794. doi: 10.1038/s42003-021-02281-0 (PMC8266802; doi:10.1038/s42003-021-02281-0)
Supplement: Supplementary file 3 — Description of Additional Supplementary Files [file 42003_2021_2281_MOESM3_ESM.pdf]

Description of Additional Supplementary Files

File name: Supplementary Movie 1

Description: Video illustrating emergent beams according to varying incidence angles. Theoretical courses of light. Notice the stability of the double reflected light when the angle of the incident light varies.

File name: Supplementary Movie 2

Description: Video illustrating emergent beams according to varying incidence angles. Experiment realized with two mirrors disposed with an angle of 148°. Spot 1, reflected light from first mirror; spot 2, reflected light from second mirror; spot 1′, stable double reflected light.
